# COX-2 Inhibition in Glioblastoma Cells Counteracts Resistance to Temozolomide by Inducing Oxidative Stress

**DOI:** 10.3390/antiox14040459

**Published:** 2025-04-12

**Authors:** Francesca Rosaria Augello, Francesca Lombardi, Valeria Ciummo, Alessia Ciafarone, Maria Grazia Cifone, Benedetta Cinque, Paola Palumbo

**Affiliations:** 1 Department of Life, Health and Environmental Sciences, University of L’Aquila, 67100 L’Aquila, Italy; francescarosaria.augello@univaq.it (F.R.A.); francesca.lombardi@univaq.it (F.L.); alessia.ciafarone@graduate.univaq.it (A.C.); mariagrazia.cifone@univaq.it (M.G.C.); benedetta.cinque@univaq.it (B.C.); 2 Department of Innovative Technologies in Medicine and Dentistry, University “G. D’Annunzio”, 66100 Chieti, Italy; valeria.ciummo@phd.unich.it

**Keywords:** glioblastoma, temozolomide, cyclooxygenase-2, inflammation, oxidative stress

## Abstract

Oxidative stress critically influences the pathophysiology of glioblastoma (GBM), a deadly and aggressive brain tumor. Reactive oxygen species (ROS) regulate cancer cell homeostasis, influencing the treatment response. The transcription factor Nuclear Factor Erythroid 2-Related Factor 2 (Nrf2) activates antioxidant defenses, protecting GBM cells from therapy-induced oxidative stress and contributing to Temozolomide (TMZ) resistance. Cyclooxygenase-2 (COX-2) plays a key role in GBM chemoresistance by modulating the tumor microenvironment and supporting a pro-survival phenotype. The impact of COX-2 inhibition by celecoxib (CXB), a selective COX-2 inhibitor, combined with TMZ on oxidative stress modulation linked to resistance was investigated in GBM primary cultures and cell lines. The drug combination CXB+TMZ was tested on TMZ-sensitive and -resistant cells, and ROS levels and Nrf2 activation were evaluated via a DCFH-DA probe and Western blotting, respectively. The oxidative stress marker malondialdehyde and antioxidant enzymes were assayed using standard methods. COX-2 inhibition combined with TMZ significantly increased ROS, while TMZ alone induced a compensatory antioxidant response, sustaining resistance. Drug combination reduced this response, restoring oxidative stress even in TMZ-resistant cells. Prostaglandin E2 reversed these effects, confirming the role of the COX-2/PGE2 axis in redox balance. Drug combination increased ROS, disrupted redox homeostasis and overcame TMZ resistance, supporting COX-2 inhibition as a promising GBM therapy strategy.

## 1. Introduction

Oxidative stress plays a dual role in cancer, promoting progression through DNA damage, inflammation, mitochondrial dysfunction, and antioxidant depletion while also inducing cellular senescence, which can exert both tumor-suppressive and pro-tumorigenic effects [1,2,3,4]. Additionally, oxidation contributes to resistance against chemotherapy and radiotherapy in tumor cells. The levels of reactive oxygen species (ROS) tightly regulate the self-renewal ability of cancer stem cells, which are primarily responsible for tumor growth and progression [5,6].

Glioblastoma multiforme (GBM) is a highly aggressive brain tumor characterized by rapid progression and resistance to standard therapies, resulting in a poor survival rate [7]. GBM is associated with a highly inflammatory tumor microenvironment rich in immune cells and mediators that promote tumor progression and therapy resistance [8].

Various studies indicate that cyclooxygenase-2 (COX-2) is overexpressed in multiple human malignant and metastatic epithelial tumors, including GBM. Research has demonstrated a clinical association between COX-2 expression and more aggressive forms of GBM, suggesting that it may be a strong predictor of poor survival outcomes [9].

A key downstream mediator of COX-2 is prostaglandin E2 (PGE2). The COX-2/PGE2 axis exerts pleiotropic effects on the tumor microenvironment by promoting immunosuppression, supporting tumor cell survival, and stem cell renewal [10]. Findings highlight the pivotal role of the COX-2/PGE_2_ signaling axis in modulating redox homeostasis and therapy resistance. Huang et al. showed that inhibiting this axis restored sensitivity to chemotherapy drug oxaliplatin in resistant colorectal cancer cells by increasing intracellular ROS levels and impairing antioxidant defenses [11]. In irradiated A549 lung cancer cells, PGE_2_, released in the tumor microenvironment, promoted the survival of neighboring non-irradiated cells by enhancing antioxidant defenses through the Nrf2-mediated upregulation of the antioxidant enzyme heme oxygenase-1 (HO-1) [12]. Similarly, in GBM, irradiation-induced COX-2 expression led to increased HO-1 levels that counteracted ROS-mediated toxicity [13].

Celecoxib (CXB) is a new-generation nonsteroidal anti-inflammatory drug (NSAID) that selectively inhibits COX-2. By reducing prostaglandin production, CXB exerts anti-inflammatory and analgesic effects. It is FDA-approved for treating several types of arthritis and managing acute or chronic pain [14,15].

Tumor cells adapt to oxidative stress by enhancing glucose metabolism to avoid cell death. However, when ROS levels exceed the antioxidant capacity of the cells, they induce genomic instability and cell death. At moderate levels, ROS act as secondary messengers that promote tumor progression by sustaining the oncogenic phenotype, driving DNA mutations and activating pro-oncogenic signaling pathways [16]. Conversely, excessive ROS levels can trigger apoptosis and cellular senescence [17]. Furthermore, ROS play a pivotal role in modulating inflammatory responses by influencing the expression of COX-2. Studies have shown that ROS are crucial activators of NF-κB, which they achieve by directly oxidizing redox-sensitive cysteines on the IκB kinase (IKK) complex, which is the principal regulator of the NF-κB signaling pathway. This activation leads to enhanced pro-inflammatory responses [18].

Chemotherapeutic agents, such as alkylating agents like Temozolomide (TMZ), the first-line chemotherapy for GBM, induce oxidative stress through the production of ROS. This process damages crucial cellular components, including DNA, proteins, and lipids, ultimately leading to cancer cell death [19,20]. However, tumor cells can adapt to ROS by upregulating antioxidant systems, allowing them to maintain redox homeostasis and survive oxidative stress. This adaptation can result in resistance to ROS-inducing therapies, presenting a significant challenge in treatment.

The transcription factor Nuclear Factor Erythroid 2-Related Factor 2 (Nrf2) is a key regulator of cellular redox homeostasis and plays a dual role in cancer biology, acting as both a tumor suppressor and an oncogene depending on the context. Under normal conditions, Nrf2 is bound to its inhibitor, Kelch-like ECH-associated protein 1 (Keap1). Once released from Keap1, Nrf2 translocates to the nucleus, binds to the antioxidant response element (ARE) in target gene promoters, and activates detoxifying and antioxidant enzymes to maintain redox balance. In response to oxidative stress, Nrf2 translocates to the nucleus and enhances intracellular antioxidant responses, including glutathione (GSH), superoxide dismutase (SOD), and catalase (CAT) [21,22]. These responses help to neutralize ROS and maintain cellular balance, promoting DNA repair and metabolic stability [23,24]. Moreover, glioma cells that stably overexpress SOD and CAT exhibit high resistance to TMZ, enhanced tumorsphere formation, and poor prognosis [25,26], and TMZ-resistant glioma cells display increased expression of CAT, SOD2, and BMI1, a protein associated with stemness and therapy resistance [27].

In cancer cells, high Nrf2 levels protect against oxidative stress, preserve redox homeostasis, contribute to cancer initiation and progression by inhibiting apoptosis in newly transformed cells, and promote resistance to therapies [23,28,29,30]. In tumors such as GBM, Nrf2 is aberrantly activated, enhancing cell survival under stressful conditions like hypoxia, chemotherapy, and radiotherapy [31,32]. Its hyperactivation enables cancer cells to evade apoptosis, increase proliferation, and resist treatment by having GSH counteract the ROS induced by therapies [24,28,33,34].

Nrf2 also promotes detoxification pathways and regulates redox-sensitive DNA repair genes such as O6-methylguanine-DNA methyltransferase (MGMT), which contribute to tumor progression and therapeutic resistance [35,36]. Interestingly, in patients with GBM treated with radiotherapy and TMZ, the presence of the concurrent methylation of both MGMT and Keap1 promoters was associated with a lower risk of tumor progression. Conversely, the methylation of MGMT when Keap1 remained unmethylated was linked to a worse prognosis. This finding supports the idea that Nrf2 activation helps protect tumor cells from damage caused by genotoxic therapies [37]. Given the pro-tumorigenic role of Nrf2 in GBM, strategies aimed at inhibiting the Nrf2 pathway could be highly beneficial.

In GBM cells, high Nrf2 expression is closely associated with resistance to TMZ treatment [28,38,39]. In studies involving U138MG and U87MG cell lines, TMZ treatment resulted in Nrf2 upregulation. Silencing Nrf2, however, restored TMZ sensitivity, leading to increased DNA damage and apoptosis [39]. Following TMZ treatment, recurrent GBM tissues exhibit nuclear Nrf2 hyperactivation, which correlates with a shorter time to tumor recurrence [40]. In TMZ-treated U87MG and U251MG cells, Nrf2 contributed to chemotherapy resistance by activating the MAPK pathway, with a dose-dependent increase in Nrf2 transcriptional activity and the phosphorylation of p38/MAPK [36]. Given Nrf2’s role in therapy resistance, Zhang and Wang demonstrated that targeting the Nrf2/ARE pathway with FTY720, a synthetic derivative of a fungal metabolite, may effectively overcome TMZ resistance in U87MG and U251MG cells. By impairing antioxidant defenses—specifically, Nrf2 and its downstream targets, HO-1 and NAD(P)H:quinone oxidoreductase-1 (NQO-1)—and promoting apoptosis, FTY720 enhances the sensitivity of GBM cells to oxidative stress and TMZ-induced cytotoxicity [41].

Nrf2 regulates more than 200 genes involved in the antioxidant response, including HO-1, a stress-inducible enzyme that breaks down heme and is activated by various stimuli [42]. High HO-1 expression in many cancers, including GBM, promotes tumor progression by leveraging the antioxidant, anti-apoptotic, and cytoprotective effects of heme degradation byproducts [43,44]. Within the tumor microenvironment, HO-1 can be expressed by both cancer cells and surrounding stromal cells, with tumor-associated macrophages (TAMs) being the primary source of HO-1. Recently, a study involving U87MG cells showed that the pharmacological inhibition of HO-1, when combined with TMZ, significantly reduced cell proliferation compared to treatment with TMZ alone [45].

Another transcription factor, Specificity Protein 1 (Sp1), is widely expressed in mammalian cells and regulates various cellular functions, including cell proliferation, differentiation, and apoptosis. Sp1 is activated by oxidative stress, and both Sp1 and Nrf2 contribute to TMZ resistance in GBM cells by enhancing antioxidant defenses and reducing oxidative damage. Chang et al. demonstrated that the Sp1-induced expression of SOD2 contributes to TMZ resistance independent of MGMT status, suggesting an alternative pathway for overcoming therapy resistance [46]. In U87MG and A172 GBM cells, Sp1 binds directly to the promoter of the SOD2 gene, which protects the cells against TMZ-mediated toxicity. This binding increases SOD2 expression and boosts the antioxidant system. Elevated SOD2 levels reduce ROS, allowing tumor cells to survive and evade apoptosis. The inhibition of Sp1 using mitromycin A restored TMZ sensitivity, as evidenced by the significant increase in ROS levels in resistant cells [46].

We recently demonstrated that TMZ can upregulate the inflammatory marker COX-2 in GBM cells that exhibit different sensitivities to TMZ. This upregulation contributes to an immunosuppressive environment and promotes the pro-tumor polarization of M2 macrophages. Interestingly, the combination of selective COX-2 inhibitors with TMZ counteracts these effects and restores the sensitivity of GBM cells to chemotherapy [47,48]. Given the role of oxidative stress in therapy resistance, our study specifically aimed to determine how COX-2 inhibition could modulate redox homeostasis in GBM cells with different sensitivities to TMZ, ultimately counteracting chemoresistance. To the best of our knowledge, this is the first study providing evidence of the impact of CXB and TMZ in a therapeutic context on oxidative stress regulation in GBM, reinforcing the potential of this drug combination as an adjunct therapy for this type of cancer.

## 2. Materials and Methods

### 2.1. Cell Lines

Human GBM grade IV cell lines, T98G and U87MG, were purchased from the European Collection of Authenticated Cell Cultures (ECACC). The main characteristics of the GBM cell lines are as follows: T98G cells are COX-2-positive and express high levels of MGMT (“TMZ-resistant”); U87MG cells are COX-2-positive and do not express MGMT (“TMZ partly resistant”) [49]. The T98G and U87MG cells were cultured in a complete medium (Dulbecco’s Modified Eagle’s Medium (DMEM) supplemented with 10% (*v*/*v*) of fetal calf serum (FCS), 2 mM L-glutamine, 100 U/mL penicillin, and 100 mg/mL streptomycin) and maintained at 37 °C in 5% CO_2_ and 95% humidity. Unless specified, all reagents and consumables were obtained from Euro Clone (West York, UK).

### 2.2. Method of Obtaining Patient-Derived Primary GBM Cultures

Tumor biopsies of GBM were obtained from two patients at the Operative Unit of Neurosurgery at the San Salvatore Hospital of L’Aquila. Each patient provided written consent in accordance with the approved ethical permit from the regional ethics board, the Internal Review Board (20 January 2015). Primary cell cultures (GL12 and GL44) were established from freshly resected biopsies, which were clinically and histologically confirmed to be GBM. They were cryopreserved at early passages as previously reported [47] and used as needed. Briefly, the specimens were mechanically cut into small pieces using a lancet and enzymatically dissociated with 0.125% trypsin and 0.125% EDTA (pH 7.4) in a DMEM serum-free solution. The ratio of GBM tissue weight to trypsin solution was 1 g/10 mL. Digestion was carried out at 37 °C for 15–20 min in a water bath with gentle stirring. The cells obtained were collected by centrifugation and grown in adherent conditions to obtain GBM primary cultures. After reaching 80% confluence, the GBM primary cultures were expanded in a complete medium.

### 2.3. Reagents and Treatments

The selective COX-2 inhibitor celecoxib (CXB), obtained from Sigma-Aldrich (Saint Louis, MO, USA), was stored in a stock solution in dimethylsulfoxide (DMSO) at −20 °C and diluted in a complete culture medium before use. Temozolomide (TMZ) (Sigma-Aldrich) was solubilized in DMSO, diluted in distilled water immediately before use, and administered at a final concentration of 51.5 mM. Based on our previous studies [47,48] and other in vitro reports on GBM [50,51], CXB (50 µM) and TMZ (100 µM for GL12 and U87MG and 200 µM for GL44 and T98G) concentrations were selected for both cell lines and GBM primary cultures. Cells treated with DMSO alone (vehicle) were used in all experiments as a “control” (CNTR). To assess the TMZ sensitivity of GL12, a dose–response curve was obtained using different TMZ concentrations (50, 100, 200, and 400 µM) for 72 h, allowing for the selection of the optimal drug concentration for subsequent experiments. For all experiments, GBM cells were seeded in 25 cm^2^ plates at 5 × 10^3^ cells/cm^2^, left to adhere overnight, and then left untreated (CNTR) or treated with TMZ or CXB alone or in combination for 24 or 72 h.

To evaluate the effects of exogenous prostaglandin E2 (PGE2) (Sigma-Aldrich), GBM cells were plated at 5 × 10^3^ cells/cm^2^, left to adhere, and then simultaneously incubated with CXB and TMZ, as previously described, and with PGE2 alone (10 µM) for 72 h.

Primary cultures were kept in culture for no longer than 2 months, and all the cell cultures were regularly checked for mycoplasma contamination using the N-GARDE Mycoplasma PCR Reagent set (EMK090020-EUC; BioCat, Heidelberg, Germany).

### 2.4. Fluorometric Measurement of Intracellular ROS Levels

Intracellular ROS levels were assessed using a DCFH-DA probe, an indicator of oxidative stress in biological systems (Immunological Sciences, Rome, Italy). In the cell, the DCFH-DA probe is deacetylated by esterase to generate nonfluorescent DCFH. In the presence of ROS, DCFH is oxidized to fluorochrome 2′,7′-dichlorofluorescein. The media of cells treated with CXB or TMZ alone or combined at the selected concentrations for 24 h were replaced with DCFH-DA solution (25 μM) at 37 °C for 30 min. ROS levels were measured using a VICTORX4™ fluorometer (PerkinElmer, Waltham, MA, USA) with excitation and emission filters of 488 and 535 nm, respectively. The values obtained were normalized for cell number and are expressed as relative fluorescence units (RFU)/10^5^ cells.

### 2.5. SOD Activity

SOD activity was evaluated in the cell lysates using an SOD assay kit (Cayman Chemical Company, Ann Arbor, MI, USA), according to the manufacturer’s instructions. CXB-, TMZ-, and CXB+TMZ-treated cells (72 h) were lysed and centrifugated at 17,949× *g* for 20 min. Then, SOD activity was evaluated using a tetrazolium salt for the detection of superoxide radicals. Absorbance was detected at 450 nm using a spectrophotometer (Bio-Rad, Hercules, CA, USA). One unit of SOD is defined as the quantity of enzyme needed to exhibit 50% neutralization of the superoxide radical (U/mL). The obtained data were normalized for total protein and are expressed in units of SOD activity/mg protein.

### 2.6. Catalase Activity

The catalase activity in the cell lysates was measured using a catalase assay kit (Cayman Chemical Company), as per the manufacturer’s instructions. The cell lysate was mixed with methanol in the presence of H_2_O_2_ to produce formaldehyde. The generated formaldehyde formed a colored product upon reacting with 4-amino-3-hydrazino-5-mercapto-1,2,4-triazole (Purpald), the concentration of which was quantified by measuring the absorbance at 540 nm with a spectrophotometer (Bio-Rad). One unit of catalase was defined as the quantity of enzyme causing the formation of 1 nmol formaldehyde per minute (nmol/min/mL). Obtained data were normalized for total protein and are expressed as units of catalase activity/mg protein.

### 2.7. Lipid Peroxidation Detection

The level of oxidative stress can be assessed by measuring various oxidative stress biomarkers, such as lipid peroxidation end products. Lipid peroxidation was investigated by measuring the levels of malondialdehyde (MDA), as a lipid peroxidation end product, in the GBM cell lysates, following 72 h of treatment with CXB or TMZ alone or in combination and PGE2, using a human MDA (ELISA) kit (Elabscience, Houston, TX, USA) according to the manufacturer’s instructions. The obtained MDA concentration values were normalized for cell number and are expressed as ng/10^5^ cells.

### 2.8. Western Blot

For Western blot analysis, cell pellets were lysed in ice-cold RIPA buffer (Merck KGaA, Darmstadt, Germany) supplemented with a 100 mM protease inhibitor cocktail (Sigma-Aldrich, Saint Louis, MO, USA). Protein concentration was assayed with the DC Protein Assay (Bio-Rad, Hercules, CA, USA). Protein lysates (25 μg) were separated on a 10% SDS–polyacrylamide gel under reducing conditions with 5% β-mercaptoethanol and electroblotted onto a 0.45 µm nitrocellulose membrane (Bio-Rad). Nonspecific binding sites on the membrane were blocked using 5% non-fat dry milk. Overnight incubation was performed at 4 °C with the following antibodies: rabbit monoclonal anti-phospho-Nrf2 (phospho-S40) 1:2000 (Abcam, Cambridge, UK), rabbit monoclonal anti-HO-1 1:2000 (Invitrogen Corporation, Waltham, MA, USA), and mouse monoclonal antibody anti-β-actin 1:1000 (Immunological Sciences, Rome, Italy). As secondary antibodies, peroxidase-conjugated anti-rabbit and anti-mouse IgG antibodies (dilution 1:5000) were acquired from Immunological Sciences. Immunoreactive band densities visualized using a chemiluminescence reagent (ECL, Thermo Fisher Scientific, Waltham, MA, USA) were quantified using the chemiluminescence documentation system ALLIANCE (UVITEC, Cambridge, UK).

### 2.9. Immunofluorescence Staining

GBM cells were grown on coverslips in a 12-well plate (seeded at 5000 cells/cm^2^) and treated as previously reported for 72 h. Successively, the coverslips were fixed with 4% formaldehyde (Carlo Erba Reagents S.r.l., Cornaredo, Italy) for 20 min, permeabilized with 0.1% Triton X-100 (Sigma-Aldrich, Saint Louis, MO, USA) for 5 min, and blocked with 3% BSA (Sigma-Aldrich) for 20 min at room temperature. Coverslips were incubated overnight at 4 °C with rabbit monoclonal anti-phospho-Nrf2 (S40) (1:250, Abcam, UK) or anti-Sp1 (1:500, Invitrogen) and then stained for 45 min at room temperature with FITC-conjugated goat anti-rabbit IgG (1:1000, Millipore, Germany). Finally, the coverslips were stained for 45 min at room temperature with TRITC-phalloidin (Sigma-Aldrich) to label F-actin. The coverslips were mounted with VECTASHIELD^®^ Antifade Mounting Medium with DAPI (Enzo Life Sciences, Lausen, Switzerland) and then examined at 100× magnification with a fluorescent microscope (Eclipse 50i, Nikon, Tokyo, Japan).

### 2.10. Statistical Analysis

All data were analyzed using GraphPad Prism version 8.0 (GraphPad Software, San Diego, CA, USA). To compare the mean values among groups, a one-way ANOVA followed by Dunnett’s or Tukey’s post hoc test was used. Data were expressed as mean ± SEM (standard error of the mean) values, as reported in figure legends. *p* values were considered statistically significant when lower than 0.05.

## 3. Results

### 3.1. Characterization of Cellular Models in Terms of Sensitivity to TMZ

First, we characterized the two patient-derived primary cultures (GL12 and GL44) by analyzing their basal levels of expression of MGMT and COX-2. To validate our findings, we also included GBM cell lines with different MGMT methylation statuses and basal COX-2 expression levels: the MGMT-negative U87MG cells and the MGMT-positive T98G cells. Our analysis confirmed that MGMT expression was high in the untreated GL44 and T98G cells, lower in the primary culture GL12, and absent in the U87MG cell line [7]. Despite the lack of MGMT expression, U87MG cells should be considered partially resistant to TMZ rather than strictly sensitive, partly due to their high level of COX-2 expression (Figure S1A). We previously assessed the effect of TMZ on GL44 and confirmed its high resistance to the treatment [47]. To assess GL12 sensitivity to TMZ cytotoxicity, cell proliferation at increasing concentrations was measured. TMZ at 50 µM had no significant effect on proliferation, whereas 100 µM led to a reduction. A bar graph of OD values reveals a dose-dependent decrease in viability from 100 to 400 µM compared to controls (CNTR) (Figure S1B). Representative microscopy images confirm a reduced cell density at higher TMZ concentrations (Figure S1B). Based on their sensitivity to TMZ and COX-2 levels, we identified the cell cultures as follows: two TMZ-resistant cultures, T98G and GL44 (MGMT^+^/COX-2^+^); one partially resistant culture, U87MG (MGMT^−^/COX-2^+^); and one TMZ-sensitive primary culture, GL12 (MGMT^−^/COX-2^+^).

### 3.2. Effect of COX-2 Inhibitor and TMZ on ROS Modulation in GBM Cells

**Figure 1:**
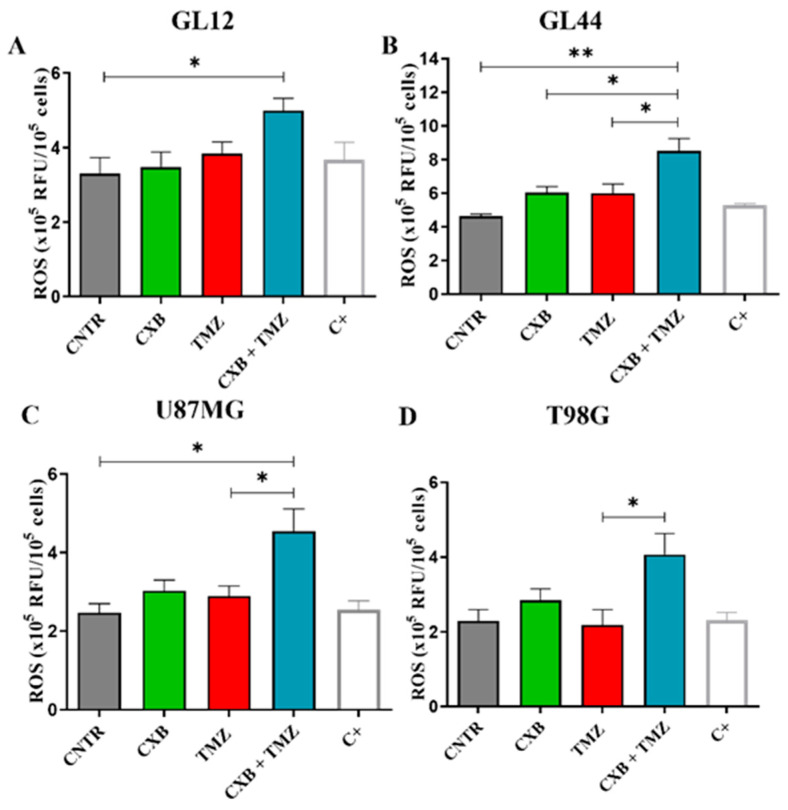
ROS measurement in patient-derived GBM primary cultures and cell lines. Intracellular ROS production was evaluated in supernatants of GL12 (**A**) and GL44 (**B**) and the cell lines U87MG (**C**) and T98G (**D**) in the absence (CNTR) and presence of CXB and TMZ and a combination of both using the 2′,7′-Dichlorodihydrofluorescein diacetate (DCFH-DA) assay after 24 h of treatment. Data are expressed as relative fluorescence units (RFU) × 10^5^ per 10^6^ cells and shown as a bar graph of three independent experiments (mean ± SEM) (* *p* < 0.05, ** *p* < 0.01).

Redox status is crucial in determining the therapeutic resistance of GBM. In this study, we tested whether the combination of the selective COX-2 inhibitor CXB with TMZ could influence ROS levels in different GBM cell models. GBM cells typically have elevated ROS levels due to imbalances in ROS production and elimination, which are primarily caused by mitochondrial dysfunction and compromised antioxidant systems [52]. To investigate the role of ROS in the TMZ resistance of GBM cells, the amount of ROS generated in patient-derived primary cultures and cell lines after exposure to CXB, TMZ, and a combination of the two drugs was measured using the DCFH-DA assay. Figure 1 presents bar graphs comparing ROS levels, measured in relative fluorescence units (RFU) × 10^5^ per 10^6^ cells, in GBM cells exposed for 24 h, as described above. A positive control (C+) is included for comparison. Previous studies have shown that basal ROS levels are higher in TMZ-resistant cells (T98G) compared to TMZ-sensitive cells (U251MG). TMZ significantly increased ROS only in U251MG [53] and in GBM primary cultures [19]. In our experiments, neither CXB nor TMZ resulted in a significant increase in ROS in any of the cultures examined when used as a single agent (Figure 1A–D). However, the drug combination notably increased ROS production in all tested GBM cells. This finding suggests a potential mechanism through which the CXB+TMZ combination increases cytotoxicity, beyond that induced by TMZ alone. In particular, in GL12 and GL44, the drug combination significantly increased ROS levels by 1.48-fold and 1.83-fold compared to the control (CNTR), respectively. Additionally, CXB+TMZ further elevated ROS levels relative to TMZ alone (1.16-fold and 1.29-fold increases in GL12 and GL44, respectively) (Figure 1A,B). The same trend was observed for cell lines: in U87MG and T98G cells, the drug combination strongly increased ROS levels by 1.76-fold and 1.78-fold versus CNTR, respectively, while TMZ alone induced 1.17-fold and 0.95-fold increases, respectively (Figure 1C,D). However, in T98G cells, the increase observed following CXB+TMZ exposure did not reach statistical significance compared to CNTR (*p* = 0.0622).

### 3.3. Different Nrf2 Levels Were Induced in GBM Cells Exposed to Treatments

**Figure 2:**
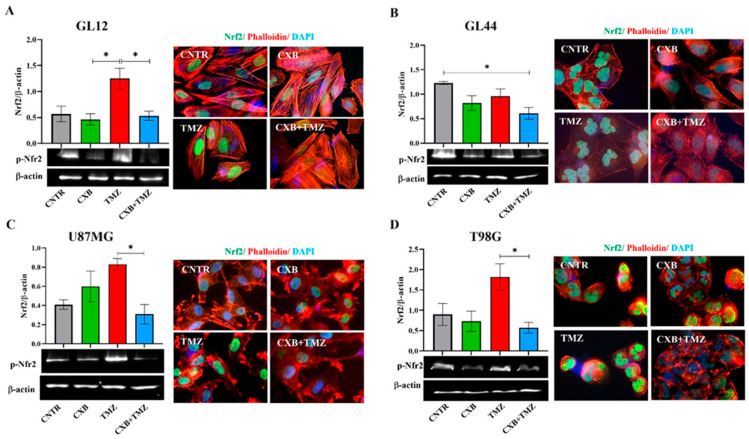
Pharmacological inhibition of COX-2 affects p-Nrf2 expression. GL12 (**A**), GL44 (**B**), and U87MG (**C**) and T98G (**D**) cells were exposed to CXB and TMZ alone or in combination for 72 h. The Western blot quantification of p-Nrf2 expression and histograms showing densitometric analysis normalized to β-actin are shown. The results were obtained from three independent experiments (mean ± SEM). A one-way ANOVA with a post hoc Tukey’s test was used (* *p* < 0.05). Immunofluorescence images show the localization of p-Nrf2 (green), actin filaments stained with phalloidin (red), and nuclei stained with DAPI (blue) in untreated cells (CNTR) and cells treated with CXB, TMZ, and a combination of the two (CXB+TMZ) (100× magnification).

Under cellular physiological conditions, ROS are balanced by an efficient antioxidant system, which neutralizes these reactive species and maintains cellular homeostasis [54]. In GL12 cells (TMZ-sensitive/MGMT-low), the basal expression of p-Nrf2 was significantly increased by TMZ treatment, while treatment with the combination CXB+TMZ led to a reduction in p-Nrf2 levels, with a significant difference compared to TMZ alone (Figure 2A). Conversely, primary culture GL44 (TMZ-resistant/MGMT-positive) cells exhibited very high p-Nrf2 levels under control conditions, suggesting that the elevated antioxidant system may contribute to their aggressive malignant phenotype. In GL44, p-Nrf2 expression after TMZ treatment did not exceed CNTR levels but was higher than that observed with the CXB+TMZ combination (Figure 2B). Consistent with previous studies, basal p-Nrf2 levels were detected in both cell lines, albeit to varying degrees [28,39]. COX-2 inhibition did not significantly affect p-Nrf2 levels compared to the CNTR cells (Figure 2C,D). Similarly to GL12, which has low MGMT levels and is TMZ-sensitive, in U87MG cells, TMZ exposure strongly upregulated p-Nrf2 compared to CXB+TMZ (Figure 2C). Notably, T98G cells behaved differently from other TMZ-resistant/MGMT+ cells such as GL44 cells, showing a response more similar to that of TMZ-sensitive cells. This difference may be due to the amplification of the NFE2L2 gene in T98G cells; this gene encodes NRF2, a key antioxidant that enhances its expression and helps to prevent intracellular ROS accumulation [28,54].

In our experiments with T98G cells, we found that TMZ alone significantly increased the levels of p-Nrf2, whereas the combination CXB+TMZ notably reduced these levels (Figure 2D). Immunofluorescence analysis revealed that p-Nrf2 was highly expressed and predominantly localized in the nuclear compartment in all GBM cellular models. Representative fluorescence images supported these findings and suggested a pro-oxidant effect of the combination of CXB and TMZ in GBM cells (Figure 2A–D).

### 3.4. Antioxidant Defense Factors in GBM Cells Following Drug Combination Exposure

**Figure 3:**
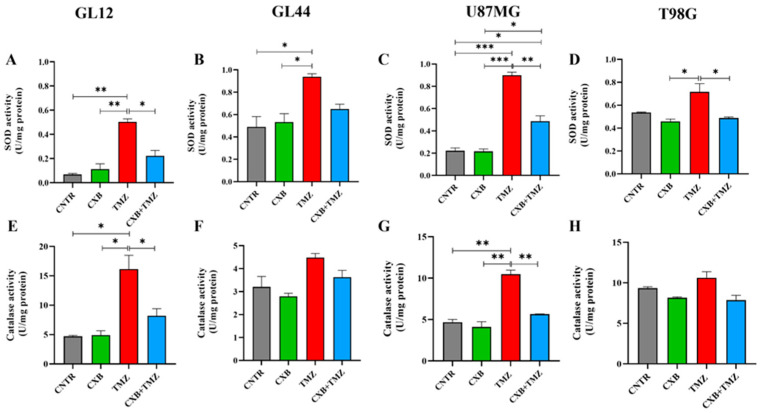
Cellular antioxidant response to the combination of CXB and TMZ. Superoxide dismutase (SOD) (**A**–**D**) and catalase (**E**–**H**) activities were evaluated in cell lysates of GL12, GL44, U87MG, and T98G, respectively, using an assay kit. The results from three independent experiments in duplicate are shown (mean ± SEM). For comparative analysis of groups of data, a one-way ANOVA with Tukey’s post hoc test was used (* *p* < 0.05, ** *p* < 0.01, *** *p* < 0.001).

Since high levels of p-Nrf2 are commonly linked to the upregulation of antioxidant response factors, we evaluated how each treatment, CXB, TMZ, or their combination, affected the antioxidant defense capacity of GBM cells. We analyzed the activities of SOD and CAT, along with the levels of HO-1 expression. Typically, SOD and CAT activity is elevated in glioma and contributes to TMZ resistance, supports tumorsphere formation, and is associated with a poor prognosis [25,26,27]. Our findings show that treatment with CXB did not significantly alter SOD and CAT activity compared to CNTR (Figure 3A–H). Consistent with p-Nrf2 levels, TMZ as a standalone treatment led to a significant increase in SOD activity across all cell cultures compared to the CNTR cells and those treated with CXB alone. This increase was also significant compared to the combination of CXB and TMZ except in the GL44 line, where a clear decrease in SOD activity was observed (Figure 3A–D). Similar results were observed for CAT activity. CXB did not affect CAT levels compared to CNTR, while TMZ induced their upregulation. Notably, the drug combination drastically reduced CAT activity only in the less TMZ-resistant cell cultures (GL12 and U87MG), whereas the decrease was not significant in the more resistant cultures (MGMT^+^/COX-2^+^), GL44 and T98G (Figure 3E–H). These findings confirm that TMZ can significantly enhance the activity of antioxidant defense enzymes in both TMZ-sensitive and TMZ-resistant cells, regardless of MGMT status. The drug combination appeared to diminish this effect.

**Figure 4:**
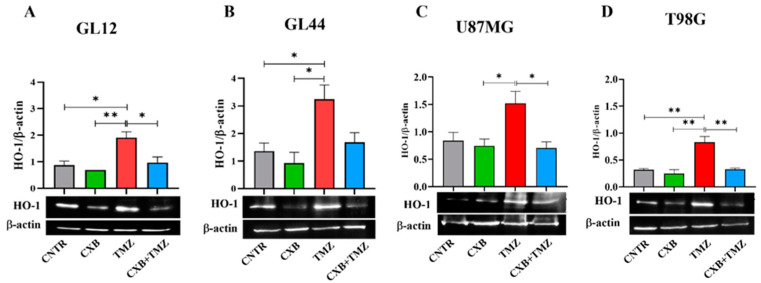
Modulation of HO-1 expression in GBM cells following CXB and TMZ treatment. Primary cultures, GL12 (**A**) and GL44 (**B**), and cell lines, U87MG (**C**) and T98G (**D**), were exposed to CXB, TMZ, and a combination of the two for 72 h, and HO-1 levels were evaluated via Western blotting. Densitometric data were normalized to β-actin. Results from three independent experiments are shown as the mean ± SEM. Statistical significance was determined with a one-way ANOVA followed by Tukey’s post hoc test (* *p* < 0.05, ** *p* < 0.01).

HO-1, an Nrf2-activated factor, protects GBM cells from oxidative stress by mitigating ROS damage and promoting resistance to therapy. We quantified the expression levels of HO-1 following treatment with CXB, TMZ, and a combination of both. In primary cultures, TMZ significantly increased HO-1 expression compared to the CNTR and CXB groups (Figure 4A,B). The combination treatment, which downregulated p-Nrf2 expression, partially reduced the TMZ-induced levels of HO-1, with significant effects observed in GL12 cells compared to TMZ alone (Figure 4A). A similar trend was confirmed in GBM cell lines (Figure 4C,D). These results strongly suggest that the antioxidant response elicited by TMZ can be reduced by COX-2 inhibition in a combination therapy approach, restoring the beneficial effects of oxidative stress in GBM cells.

### 3.5. Modulation of Oxidative Stress and Sp1 Levels by Exogenous PGE2 in GBM Cells: Implications for TMZ Resistance

**Figure 5:**
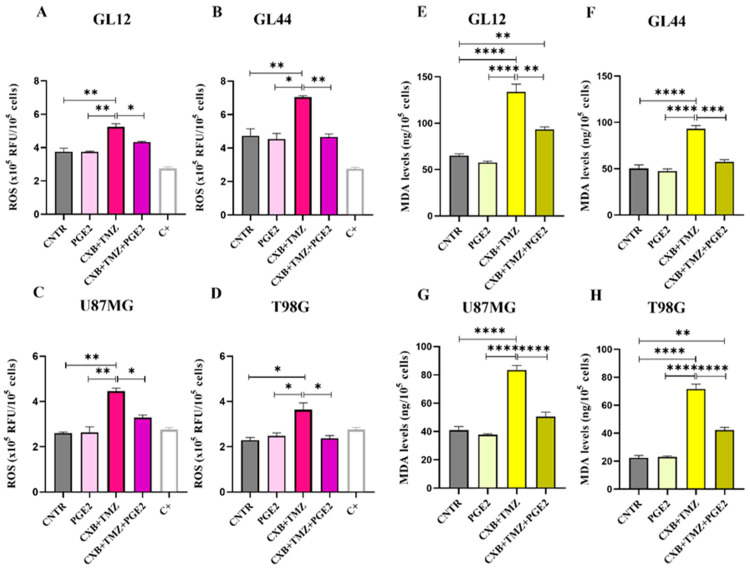
Exogenous PGE2 restored resistance to TMZ in GBM cells. ROS evaluation in the supernatants of GL12 (**A**), GL44 (**B**), U87MG (**C**), and T98G (**D**) cells after 24 h of stimulation with exogenous PGE2 and the drug combination CXB+TMZ. Values are shown as the mean ± SEM of three independent experiments. (**E**–**H**) Representative histograms of lipid peroxidation evaluated using MDA levels in GL12 (**E**), GL44 (**F**), U87MG (**G**), and T98G (**H**) cells treated with PGE2, CXB+TMZ alone, and the two drugs in combination. The results are shown as the mean ± SEM of three independent experiments. A one-way ANOVA with a post hoc Tukey’s test was used (* *p* < 0.05, ** *p* < 0.01, *** *p* < 0.001, **** *p* < 0.0001).

To clearly establish the crucial role of the COX-2/PGE2 axis in modulating oxidative stress caused by TMZ and its involvement in GBM chemoresistance, we assessed the impact of exogenous PGE2 on the GL12, GL44, U87MG, and T98G cell lines. This evaluation was conducted following treatment with the combination of CXB and TMZ (Figure 5). As previously shown in Figure 1, the drug combination significantly increased ROS levels in all the tested cell lines. Notably, the addition of PGE2, combined with CXB and TMZ, completely reversed the ROS increase induced by the drug combination, reducing ROS levels to those observed in the control group across both primary cultures and cell lines (Figure 5A–D).

To further confirm the oxidative stress-induced damage in GBM cells post-treatment, we measured the levels of MDA, which is a biomarker of cellular oxidation and a highly reactive byproduct of lipid peroxidation, a process induced by ROS that leads to decreased membrane fluidity, increased permeability, and damage to membrane proteins [55]. The elevated ROS levels induced by CXB+TMZ, as shown in Figure 1, caused MDA accumulation, suggesting possible membrane damage in all cell lines (Figure 5E–H). The addition of PGE2 dramatically counteracted the effect of CXB+TMZ, reducing the MDA levels to values similar to those observed in the CNTR cells and always significantly different from CXB+TMZ-treated cells in both patient-derived cultures and cell lines (Figure 5E–H). Therefore, PGE2 seems to mitigate the effect of the CXB+TMZ combination, suggesting a potential protective role of COX-2 against lipid peroxidation.

**Figure 6:**
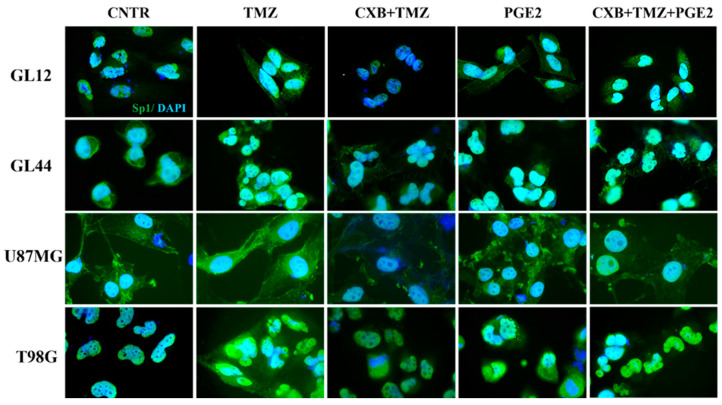
PGE2 addition restored the Sp1 expression suppressed by the drug combination in GBM cells. Immunofluorescence staining of GL12, GL44, U87MG, and T98G shows the localization of Sp1 (green), and nuclei stained with DAPI (blue) in untreated cells (CNTR) and TMZ-, CXB+TMZ-, PGE2-, and CXB+TMZ+PGE2-treated cells. All images were acquired at 100× magnification.

Considering Sp1’s role as a stress-sensitive transcription factor that enhances the expression of genes that protect against cellular damage [56], we examined its levels after treatment with exogenous PGE2 and the drug combination. Figure 6 shows immunofluorescence staining images of GBM cells treated as described, highlighting the localization of the Sp1 transcription factor (green fluorescence), which was primarily found within the nuclei. Consistent with data from the existing literature [46], we observed higher levels of Sp1 following TMZ exposure, particularly in resistant cell lines (GL44 and T98G). In contrast, exposure to CXB+TMZ suppressed Sp1 expression. Notably, elevated Sp1 fluorescence was also detected after PGE2 treatment. The addition of exogenous PGE2 to the drug combination restored Sp1 levels to those observed with TMZ alone. Despite the varying intrinsic characteristics of the tested cell cultures, such as MGMT methylation status and their subsequent sensitivity to TMZ, these findings indicate a significant involvement of COX-2 in Sp1 induction (Figure 6).

## 4. Discussion

Our study highlights the complex relationship between ROS production, antioxidant defense, and chemotherapy response in GBM cells, which exhibit varying degrees of sensitivity to TMZ. We provide compelling evidence that COX-2 inhibition with CXB, in combination with TMZ, influences oxidative stress by affecting key redox-related pathways, including the Nrf2/HO-1 axis and Sp1-mediated antioxidant responses. We confirmed that TMZ alone enhanced antioxidant defense pathways in GBM cells, resulting in the upregulation of Nrf2 and its downstream targets, SOD, CAT, and HO-1. This finding is consistent with previous research, suggesting that the overactivation of Nrf2 contributes to therapy resistance by promoting redox homeostasis and preventing apoptosis [57,58].

Given that GBM heavily relies on Nrf2 for cytoprotection through detoxifying enzymes, antioxidant defense, and metabolic reprogramming [59], Nrf2 inhibitors show significant therapeutic potential. However, there are currently no FDA-approved drugs available targeting Nrf2 [60]. Here, we tested the combination of CXB and TMZ as a potential strategy capable of targeting Nrf2. Combining drugs is an increasingly common strategy that enhances anti-tumor efficacy while allowing for lower doses of each individual drug. Interestingly, our data reveal that combined treatment with CXB and TMZ significantly increased ROS levels in GBM cells, leading to a reduction in p-Nrf2 expression and detoxification-related genes in both primary GBM cultures and cell lines, regardless of their MGMT status. This finding highlights the pro-oxidant effect of this treatment in both TMZ-resistant and TMZ-sensitive cells, supporting the hypothesis that COX-2 inhibition modulates the antioxidant response. As a result, the reduced ability to counteract oxidative damage enhances sensitivity to TMZ-induced stress. Notably, the results obtained in the GL44 primary culture slightly differ from those of other cell models in terms of significance. This may be due to its higher malignancy, attributed to elevated baseline levels of COX-2 and MGMT. Using this patient-derived culture has helped us better investigate and recapitulate the extreme heterogeneity of GBM.

Therefore, in addition to its role in COX-2 inhibition, CXB exhibits a new anticancer function. By exacerbating oxidative stress, the combination of CXB and TMZ could potentially restore the cytotoxic effects of TMZ. To support our hypothesis regarding the pro-oxidant effect of the drug combination, we cite the relevant literature addressing this subject, even if it does not directly relate to GBM. Ralph et al. described the pro-oxidant functions of NSAIDs, including CXB, emphasizing that their anticancer effectiveness is not primarily due to the inhibition of COX-2. While NSAIDs are commonly recognized for their ability to inhibit COX-2, their anticancer activity appears to be primarily driven by alternative mechanisms, including the induction of oxidative stress [61]. One of CXB’s anticancer activities is its ability to directly inhibit mitochondrial respiration, disrupt transmembrane potential, and reduce ATP production at high concentrations. This inhibition leads to excessive superoxide generation as a byproduct of the electron transport chain, ultimately triggering caspase activation and inducing tumor cell apoptosis [62]. Furthermore, Pritchard et al. examined the effects of CXB, revealing that even at lower concentrations, it interferes with mitochondrial metabolism and the survival of metastatic cancer cells. They found that CXB inhibited mitochondrial oxygen consumption, which is part of the mitochondrial respiratory pathway, leading to decreased ATP production and the excessive accumulation of ROS. This condition triggered caspase activation, resulting in apoptotic cell death. The study highlighted that CXB’s cytotoxic effect appears to be independent of COX-2 inhibition, suggesting an alternative mechanism of action centered on mitochondrial dysfunction and oxidative stress in metastatic cancer cells [63]. Zhu et al. demonstrated that CXB can enhance ROS in cutaneous squamous cell carcinoma (SCC) cells, disrupting redox homeostasis and inducing oxidative stress, which impairs cancer cell survival [64]. SCC cells treated with CXB showed increased sensitivity to apoptosis triggered by death ligands such as TNF-related apoptosis-inducing ligand (TRAIL). This increased sensitivity is likely due to ROS-mediated pathways that enhance pro-apoptotic signaling, potentially leading to mitochondrial dysfunction or apoptosis modulation. Laube et al. emphasized the dual role of CXB in radiotherapy. It acts as an antioxidant to protect normal tissues while simultaneously serving as a pro-oxidant that sensitizes gliomas to radiation therapy. In tumor cells, CXB increases ROS levels, inducing apoptosis, potentially through mitochondrial dysfunction and the modulation of redox signaling. In contrast, its antioxidant effect in normal cells may arise from mitochondrial stabilization and the enhancement of endogenous defenses [65].

Unlike previous works that underline COX-2-independent mechanisms as key drivers of CXB’s pro-oxidant effects, our results show that CXB’s anti-tumor activity is COX-2-dependent and were further confirmed by the addition of exogenous PGE2. To ultimately confirm COX-2’s involvement in redox modulation and drug resistance, experiments using the COX-2-negative U251MG GBM cell line were performed (Figure S2). In these cells, treatment with CXB, TMZ, or their combination failed to significantly alter ROS production, Nrf2 phosphorylation, the expression levels of Nrf2, and the activity of key antioxidant enzymes SOD, catalase, and HO-1 (Figure S2A–E). In contrast to COX-2-positive cell cultures, no activation of antioxidant defense pathways nor restoration of redox imbalance was observed. Thus, the presence of basal and/or TMZ-induced COX-2 in GBM cells promotes the activation of protective antioxidant responses, enhancing resistance to TMZ-induced oxidative stress.

Moreover, COX-2 inhibition effectively downregulated Sp1 levels, potentially impairing GBM cells’ ability to manage oxidative stress and survive TMZ-induced stress. Additionally, the ability of PGE2 to restore Sp1 expression supports its role in TMZ resistance, likely by enhancing the antioxidant defense system through the upregulation of SOD2. These findings suggest that COX-2-derived PGE2 plays a crucial role in maintaining oxidative stress balance in GBM cells, providing an alternative mechanism for evading chemotherapy-induced ROS-mediated damage. Furthermore, the addition of exogenous PGE2 completely countered the pro-oxidant effects of the combination of CXB and TMZ, restoring redox homeostasis and Sp1 expression.

**Figure 7:**
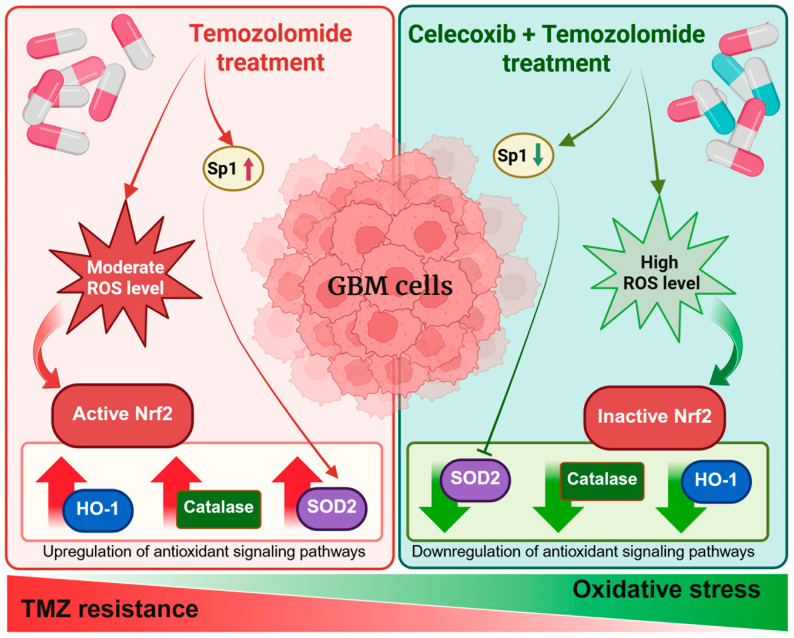
Schematic overview of the experimental approach and key findings.

COX-2 acts as a critical mediator that supports chemoresistance in GBM by sustaining antioxidant defenses and limiting oxidative stress-induced cytotoxicity. A graphical summary illustrates the main findings of this study, highlighting how the CXB and TMZ combination counteracts therapy resistance in GBM cells by enhancing oxidative stress. Specifically, COX-2 inhibition prevents the activation of antioxidant responses mediated by Nrf2 and Sp1, ultimately impairing the redox balance and sensitizing tumor cells to TMZ (Figure 7).

## 5. Conclusions

The combination of a COX-2 inhibitor and TMZ acts on GBM cells, not only inducing cell death regardless of MGMT status or baseline COX-2 levels but also addressing oxidative stress. Additionally, in cells treated with TMZ, COX-2 inhibition results in the downregulation of key antioxidant pathways, including the Nrf2/HO-1 axis and Sp1-mediated responses. This suggests that targeting COX-2 may be a promising therapeutic strategy of enhancing GBM sensitivity to TMZ. Therefore, the concurrent use of CXB and TMZ should be encouraged. A limitation of this study is its reliance on 2D cell cultures. We recognize that these culture conditions can affect cellular responses to drugs and may influence the cytotoxicity results observed. We are currently planning experiments using 3D models that include both GBM and immune cells. This approach will better mimic the tumor microenvironment and more accurately reflect cell–cell and cell–extracellular matrix interactions. Future investigations will explore how these drugs, alone or in a combination approach, affect GBM cell energy metabolism through the direct assessment of mitochondrial function and metabolic activity.

## Data Availability

The original contributions presented in this study are included in the article/Supplementary Materials. Further inquiries can be directed to the corresponding author.

## Supplementary Materials

The following supporting information can be downloaded at: https://www.mdpi.com/article/10.3390/antiox14040459/s1, Figure S1: (A) MGMT and COX-2 basal expression in GBM primary cultures (GL12 and GL44) and GBM cell lines (U87MG and T98G). Representative Western blot images of MGMT and COX-2 in GBM cells, with β-actin as the loading control. (B) The response of a GL12 primary culture to increasing concentrations of TMZ (50–400 µM) in terms of cell viability after 72 h was assessed using the CCK8 assay. Data are from three independent experiments conducted in triplicate (mean ± SEM). A one-way ANOVA followed by Dunnet’s post hoc test was used (** *p* < 0.01, **** *p* < 0.0001 vs. CNTR). Representative images of TMZ-treated GL12 cells are presented (10× magnification); Figure S2: Effect of CXB+TMZ on oxidative stress in U251MG GBM cell line. U251MG cells were treated with CXB, TMZ, and a combination as described. (A) Intracellular ROS levels were assessed using the DCFH-DA assay after 24 h of treatment. The results from three independent experiments are shown as the mean ± SEM. (B) p-Nrf2 and (C) HO-1 levels were evaluated by Western blot. Data obtained by densitometric analysis were normalized to β-actin. The results from three independent experiments are shown as the mean ± SEM. (D) SOD and (E) catalase activities were evaluated in cell lysates using assay kits. The results from three independent experiments in duplicate are shown as the mean ± SEM. For comparative analysis of groups of data, a one-way ANOVA with Tukey’s post hoc test was used.

## Abbreviations

The following abbreviations are used in this manuscript:

GBM	Glioblastoma
TMZ	Temozolomide
COX-2	Cyclooxygenase-2
CXB	Celecoxib
ROS	Reactive oxygen species
NSAID	Nonsteroidal anti-inflammatory drug
Nrf2	Nuclear factor erythroid 2-related factor 2
SOD	Superoxide dismutase
CAT	Catalase
MGMT	O6-methylguanine-DNA methyltransferase
HO-1	Heme oxygenase-1
Sp1	Specificity protein 1
DMSO	Dimethylsulphoxide
PGE2	Prostaglandin E2
MDA	Malondialdehyde
PBS	Phosphate-buffered saline
TAMs	Tumor-associated macrophages
CNTR	Control
ANOVA	Analysis of variance
