# Supplementary material for: COX-2 Inhibition in Glioblastoma Cells Counteracts Resistance to Temozolomide by Inducing Oxidative Stress
Source: Antioxidants (Basel). 2025 Apr 12;14(4):459. doi: 10.3390/antiox14040459 (PMC12024373; doi:10.3390/antiox14040459)
Supplement: Supplementary file 1 [file antioxidants-14-00459-s001.zip › antioxidants-3552937-supplementary.pdf]

Supplementary figures

COX-2 Inhibition in Glioblastoma Cells Counteracts Resistance to Temozolomide by Inducing Oxidative Stress

Francesca Rosaria Augello, Francesca Lombardi, Valeria Ciummo, Alessia Ciafarone, Maria Grazia Cifone, Benedetta Cinque, Paola Palumbo

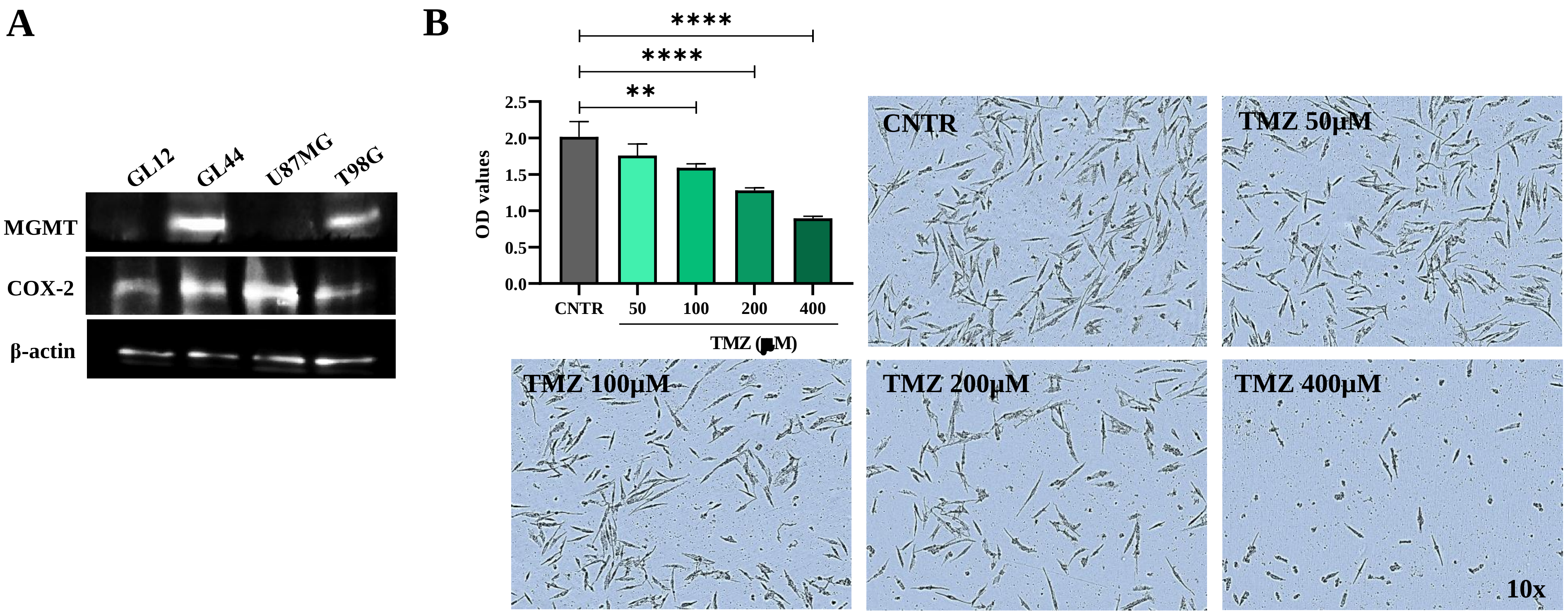

Figure S1. (A) MGMT and COX-2 basal expression in GBM primary cultures (GL12 and GL44) and GBM cell lines (U87MG and T98G). Representative Western blot images of MGMT and COX-2 in GBM cells, with β-actin as the loading control. (B) The response of GL12 primary culture to increasing concentrations of TMZ (50–400 µM) in terms of cell viability after 72 hrs was assessed using the CCK8 assay. Data are from three independent experiments in triplicate (mean ± SEM). One-way ANOVA followed by Dunnet post-hoc test was used (**p<0.01, ****p<0.0001 vs. CNTR). Representative images of TMZ-treated GL12 cells were presented (10× magnification).

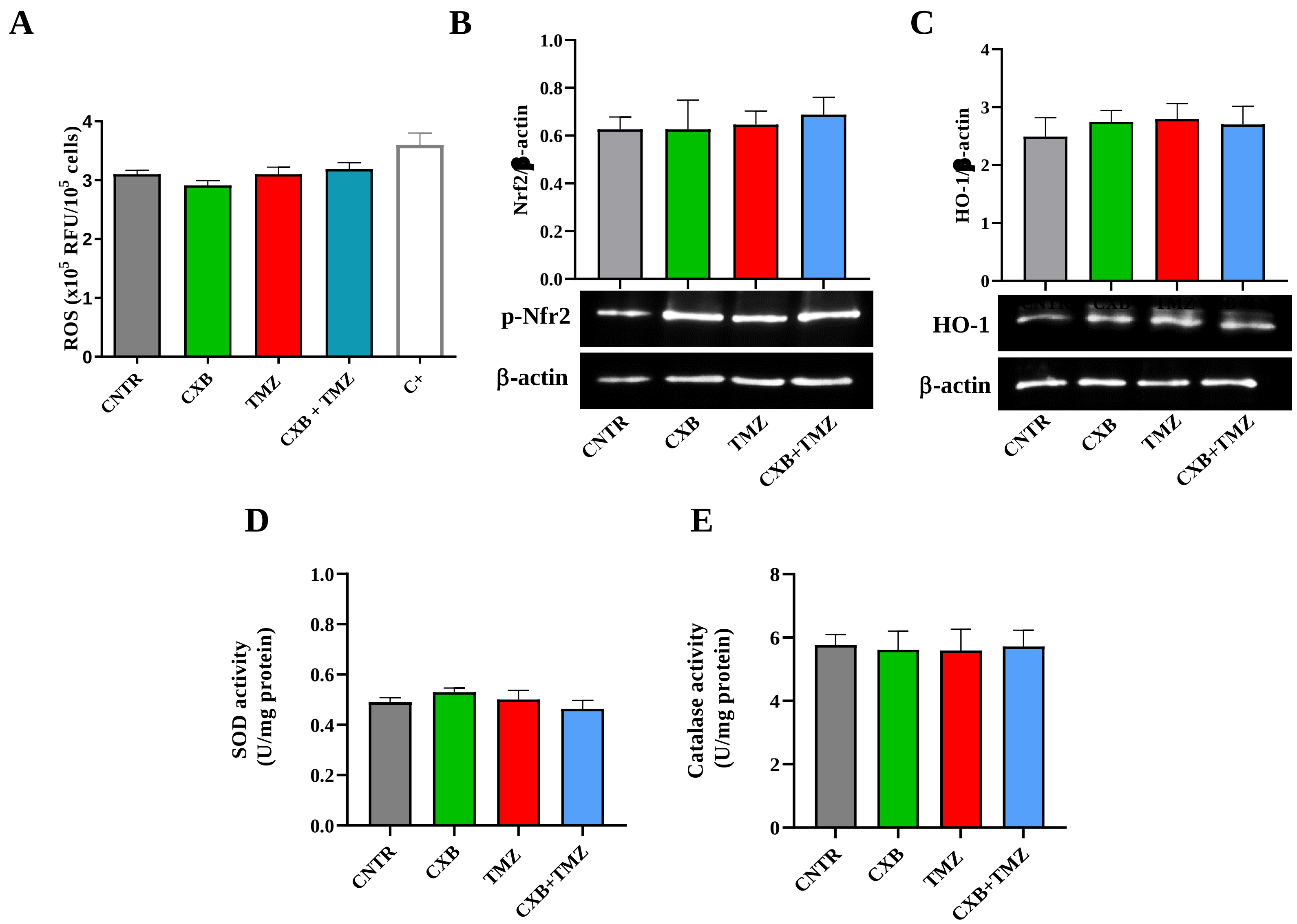

Figure S2. Effect of CXB+TMZ on oxidative stress in U251MG GBM cell line. U251MG were treated with CXB, TMZ and combination as described. (A) Intracellular ROS levels were assessed using the DCFH-DA assay after 24 hrs of treatment. The results from three independent experiments are shown as the mean ± SEM. (B) p-Nrf2 and (C) HO-1 levels were evaluated by western blot. Data obtained by densitometric analysis were normalized vs. to β-actin. The results from three independent experiments are shown as the mean ± SEM. (D) SOD and (E) catalase activities were evaluated in cell lysates using assays kit. The results from three independent experiments in duplicate are shown as the mean ± SEM. For comparative analysis of groups of data, the one-way ANOVA with Tukey post hoc test was used.
